# Wind Instruments and Oral Health: Challenges Faced by Professional Wind Musicians

**DOI:** 10.3390/dj12100306

**Published:** 2024-09-26

**Authors:** Nils P. Czech, Kurt W. Alt

**Affiliations:** Centre of Natural and Cultural Human History, Danube Private University, 3500 Krems-Stein, NÖ, Austria

**Keywords:** oral health, oral medicine, temporomandibular joint disorders, preventive health services, dentist–patient relations, music

## Abstract

Background: Recent studies have shown an association between playing wind instruments and their impact on the orofacial system. However, they have not fully evaluated all aspects of the topic, leaving a gap in the overall understanding. Methods: A thorough search of the National Library of Medicine database was conducted using our research strategy, resulting in the identification of relevant studies. An expert perspective was obtained by conducting two in-depth expert interviews with a professor of horn-playing and a specialised dentist. Results: Thirty-seven relevant publications were included in the traditional literature review. The most common diseases among professional wind instrumentalists include the lip area, temporomandibular joint, oral mucosa, respiratory system, oral allergic reactions, and orofacial trauma. Special measures, preventive measures, and expert opinions were utilised to address and overcome the associated orofacial problems. Conclusions: Wind instruments affect the oral health and tooth movement of professional instrumentalists, and dentists should consider the impact of dental changes on embouchure and performance. Dental impressions and three-dimensional intra-oral scans are important for reconstruction. This research highlights the need for specialised dental care for professional wind instrumentalists, and further studies are necessary to fully explore this topic.

## 1. Introduction

Professional wind instrumentalists frequently encounter dental-related health issues throughout their careers, primarily due to the intricate demands placed on their oral structures. Their mouth is crucial to their performance, as the tongue, teeth, lips, and cheeks work in delicate harmony during the embouchure [1,2]. Even minor disruptions in this coordination can severely impact an instrumentalist’s ability to perform, affecting both embouchure and sound quality. Orthodontic treatment and certain dental conditions, such as root resorption, orofacial trauma involving the teeth, or increased mobility of the anterior teeth, can significantly affect an instrumentalist and may even lead to occupational disability [3,4].

The technique required to play a wind instrument is highly specific, with each instrument demanding unique pressure, breath flow, and lip positioning to achieve the desired pitch and sound quality [5]. For instance, brass instrumentalists use a bowl-shaped mouthpiece that is pressed against the lips, while single-reed instrumentalists use a wedge-shaped mouthpiece that partly enters the oral cavity [6]. These activities subject the central incisors to significant mechanical stress, which, if excessive, can lead to tooth movement, protrusion, and increased mobility [7,8].

The impact of these demands is not limited to adults; children who begin learning wind instruments at an early age (typically between 4 and 8 years old) are also affected [9]. The continuous practice required to achieve professional status can influence tooth position and occlusion, particularly given the forces exerted on the anterior teeth [10]. Additionally, orofacial trauma is more common in children aged 10–14 years compared to adults [11]. Both factors are significant as they affect the technique and positioning of the mouthpiece during the embouchure.

As adults, wind instrumentalists are prone to a variety of oral diseases commonly found in the general population, such as caries, periodontal diseases, and malocclusions. While these oral health issues are generally not serious or life-threatening, they can pose significant challenges for musicians [12,13]. Given the critical role of the lips and teeth in performance, irregularities such as malocclusions, periapical inflammation, or sharp edges on fillings and crowns can be painful and limit performance [2]. Insufficient prosthetic restorations or extensive orthodontic treatments may interfere with the precise use of the mouthpiece [14]. Any dental and orofacial deficiencies that disrupt embouchure and habitual movements are problematic for professional wind instrumentalists.

Wind instrumentalists may require treatment from dentists across six sub-disciplines: oral and maxillofacial surgeons, orthodontists, endodontists, prosthodontists, periodontists, and paediatric dentists. However, finding a dentist who understands the unique needs of wind musicians and can provide treatment without compromising their embouchure can be challenging. After years of playing, each musician develops a unique habitual muscular pattern, and currently, there is no reliable tool to measure embouchure [7,15]. Most dentists lack the specialised diagnostic tools and expertise needed to address the specific oral health problems of wind instrumentalists. Only a small number of dentists worldwide specialise in treating professional musicians.

This scoping review included original articles, case studies, and review articles. The primary aim of this study is to raise awareness among dentists about the diagnosis, treatment, and preventive measures essential for the care of professional wind instrumentalists. Given the critical role of the embouchure in a musician’s performance, all dental treatments for this group must prioritise the preservation of these delicate oral structures. This article focusses on the orofacial structures and health problems unique to wind instrumentalists, highlighting the importance of specialised knowledge for dental practitioners.

## 2. Methods

### 2.1. Research Strategy

The research was conducted using the National Library of Medicine’s database via PubMed and ResearchGate to retrieve relevant articles in English and German on oral health issues in wind instrumentalists. Articles were studied in depth and sorted by relevance into five categories: embouchure, health problems, dentistry, temporomandibular disorders, and the respiratory system. Only full-length articles on oral health in wind instrumentalists were selected; other instruments were excluded. Papers on general health problems were included only if they focused on orofacial issues in wind instrumentalists.

**Figure 1:**
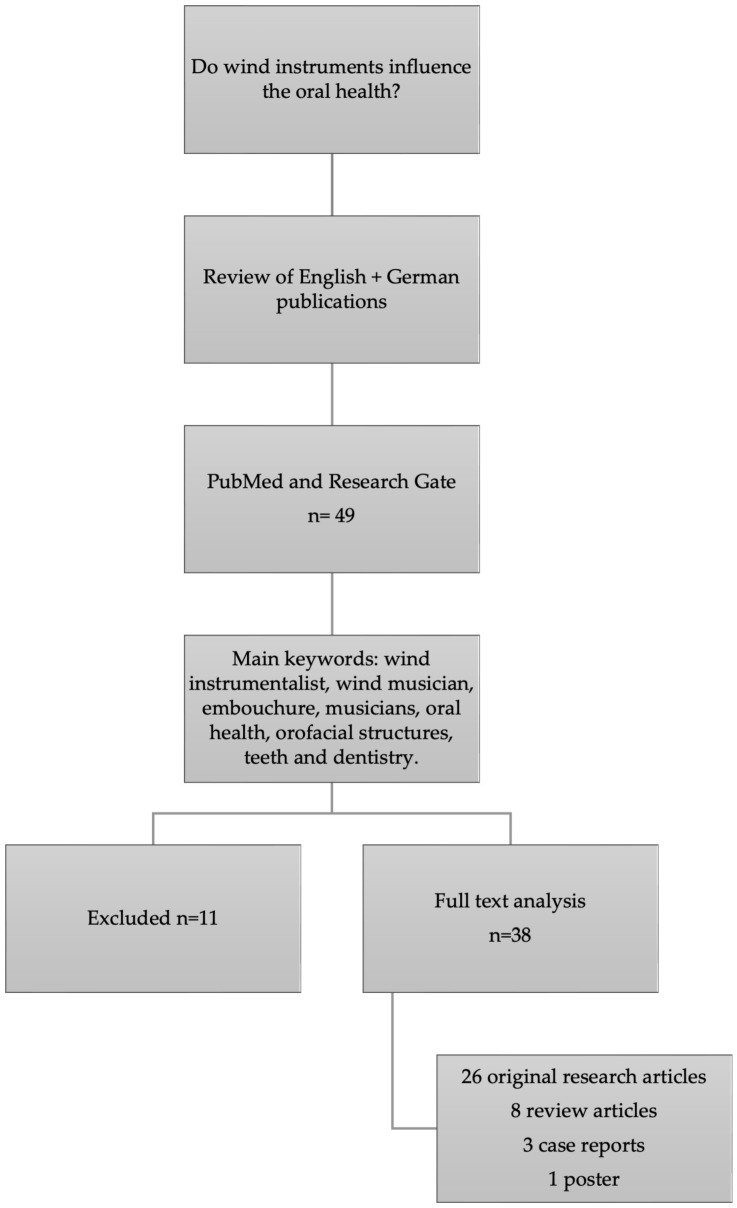
Flowchart of search and selection.

Using this strategy, 49 articles were identified (Figure 1). Some were excluded for lack of relevance, resulting in 38 included articles: 26 original research articles, eight review articles, three case reports, and one poster. Currently, previous articles on this topic are limited and focus on specific questions in modern dentistry, such as temporomandibular disorder (TMD), and do not address the whole topic [16,17]. More than half of the articles were published within the last 10 years.

### 2.2. Study Selection

After extensive evaluation, all articles were sorted, listed, and summarised in Supplementary Table S1, which highlights the key findings for each aspect of the topic. Our research article addresses various orofacial health issues among wind instrumentalists and targets two groups of readers. We primarily targeted dentists, as few are familiar with the specific clinical symptoms of wind instrumentalists. Additionally, we aimed to educate musicians about typical occupational diseases and where to seek professional help from specialised dentists.

### 2.3. Sex Ratio in the Studied Groups

Generally, the sex ratio among wind instrumentalists favours males. This trend is influenced by factors like females’ preference for different instruments, sociocultural or religious reasons, and physiological limitations, as noted in a Nigerian study [18]. Some females were excluded from studies for physiological reasons [19], resulting in some ensembles being all-male. Most orchestras aim for optimal performance and sound rather than gender balance. Additionally, some studies excluded females due to their low representation, and others did not mention sex distribution as it was irrelevant to their outcomes. However, most articles presented a balanced sex ratio.

### 2.4. Sample Sizes

The sample size differed greatly depending on the study. The largest sample size was obtained from van Selms et al. (2020), who collected data using questionnaires from 1470 musicians [20]. In contrast, the case studies by van der Weijden, Berkhout, and Lobbezoo (2019) and Clemente et al. (2018, 2020) were performed on a professional instrumentalist [21,22,23]. The scientific reviews included 10–221 articles in their respective studies. 

### 2.5. Musicians Background

Musicians assessed were categorised as either amateur or professional wind musicians. Most professionals were orchestra members, had over 10 years of experience, held a master’s degree in music, or taught at a university. Amateurs included high school students, children, or individuals with 1–2 years of practice. Some studies assessed both amateur and professional musicians, but most focused on professional wind instrumentalists.

### 2.6. Instrument Selection

The range of instruments varied by group and study. Different ensembles, such as symphony orchestras and brass bands, play a variety of instruments. Common wind instruments include trumpets, tubas, saxophones, oboes, clarinets, bassoons, French horns, and flutes. Occasionally, string instrumentalists were part of an ensemble or orchestra and included in the study. Some authors focused on specific wind instruments, but all studies centred on wind instrumentalists using mouthpieces or reeds.

## 3. Results

### 3.1. Study Characteristics

This chapter examines various aspects of the oral health (in the soft and hard tissues) of professional wind instrumentalists, including the lips, maxillary incisors, allergic reactions of the skin and mucosa, TMD, respiratory system, orofacial trauma, precautions during dental treatment, and general preventive measures. 

### 3.2. The Indispensable Role of the Lips: The Heart and Brain of a Wind Musician

The lips serve as the main sensory organ for instrumentalists, providing feedback on the embouchure and functioning as an airtight seal. The friction and vibration of the mouthpiece often lead to painful, dry lips with small injuries and soft tissue trauma [14,24]. Mauersberger (2016) studied the lips of 50 brass instrumentalists after an hour of playing [25]. Of these, 73% had inconspicuous lips, 26% showed morphological abnormalities, and 12% had irreversible scar tissue on the upper lip. One player had a large upper lip scar due to previous trauma, resulting in increased air consumption and inadequate lip tension, ultimately ending his professional career. Dentists should recognise muscle dystonia and nerve pressure damage in wind instrumentalists as occupational diseases [25]. As regular examiners of the oral cavity and surrounding soft tissues, dentists are well-positioned to identify morphological changes and provide guidance to wind instrumentalists.

### 3.3. The Purpose of the Teeth: Central Workpiece for Positioning and Safety during Play

Teeth are essential for playing wind instruments, whether natural, ceramic, or plastic, as musicians use their teeth to position instruments and direct air into the mouthpiece. Maintaining good oral health is crucial for wind instrumentalists due to their reliance on teeth and orofacial structures. A two-year study by Herman et al. (1981) found significant anterior tooth movement in 11–13-year-old wind players. Cup-shaped brass mouthpieces reduced overbite and overjet, while single-reed mouthpieces increased them [10]. Further studies have also found that facial morphology and tooth position are significantly influenced in children and adults by playing wind instruments [6,13]. For instance, a longitudinal study by Barbieri (2020) showed that brass and single-reed players had slower growth in anterior facial height from ages 6 to 15 and thicker lips compared to controls. A meta-analysis found a notable reduction in overjet in children playing brass instruments over 6 months to 3 years.

Constant force over time aggravates tooth movement. For instance, 35–60 g of force applied for 6 h can result in measurable orthodontic tooth movement [26]. Fixed orthodontic appliances can generate up to 100 g of force [27]. Professional wind instrumentalists often practice for 6–8 h a day. Studies found that wind instruments exert greater forces on teeth than typical facial muscle contractions [14]. Two recent studies evaluated the forces exerted on the anterior teeth in reed instrumentalists using piezoresistive sensors. Clemente et al. (2019) examined five clarinettists screening for a medium force on the incisors using a mouthpiece of 16–120 g compared to 5–169 g in five saxophonists [1]. The author further examined the participants and found that in a 30-year-old clarinettist, high-pitched notes can exert a force of up to 379 g on tooth 21 and 88 g on tooth 11, exceeding the minimum force of 35–60 g required for orthodontic tooth movement. In comparison, low-pitched notes produced the least force on the anterior teeth. Most clarinettists stabilise their instruments using tooth 21, as it is the most comfortable during embouchure. Several studies have proven that wind instruments can produce sufficient unphysiological forces for tooth movement [27].

Studies have shown that playing wind instruments can support intentional orthodontic tooth movement in cases of malocclusion [10,14,27]. Strayer (1939) categorised wind instruments into four types based on the mouthpiece, recommending their use for specific malocclusions: Class A (cup-shaped) for hypotonic Class II cases, Class B (single-reed) for Class III cases, Class C (double-reed) for hypertonicity, and Class D (aperture) for Class I and III cases with a short upper lip [2]. Long-term studies have shown a reduction in overjet among child brass players [8,10]. A 2-year study by Herman (1981) on 11- to 13-year-olds found that playing certain musical instruments led to significant anterior tooth movements, suggesting that dentists could recommend instruments to help manage overjet and overbite. Additionally, playing wind instruments is associated with decreased anterior facial height and wider dental arches, likely due to increased orofacial muscle activity and intra-oral pressure [8]. Gualtieri (1979) found that adult single-reed players had larger overjets than controls [28]. Buccal crossbite development is more common in brass players using large cup-shaped mouthpieces [29].

Furthermore, a study by Adeyemi and Otuyemi [18,30] revealed that wind instruments affected maxillary anterior tooth alignment but not occlusal characteristics. No difference was found between Class A and B instrumentalists regarding overbite and overjet, likely because musicians rest their mouthpieces on the upper lip, applying more force to the upper incisors. Further studies have shown that wind instruments did not significantly affect the dental arch dimensions, nor did the type of instrument, duration, or frequency of playing [30,31]. 

Although more long-term research is needed, a lip pressure appliance made of 1-mm thermoformable ethylene-vinyl acetate can reduce forces on teeth during play with minimal impact on sound quality, aesthetics, or comfort [1,23].

Orthodontic treatment can also improve the embouchure of professional wind instrumentalists. Van der Weijden, Berkhout, and Lobbezoo (2019) reported a case of a professional horn player whose performance improved after lateral incisor alignment in the upper and lower jaw [21]. Composite veneers corrected the lateral tooth irregularity, followed by orthodontic alignment of the lower incisors over three months. This eliminated the patient’s lower lip pain and created a smoother vibration plane. Post-treatment, the horn player stated he would encourage his son to undergo orthodontic corrections for potential benefits. However, many wind musicians avoid such treatments as they can take years and obstruct playing. Upper retainers and buccal brackets are particularly uncomfortable, causing pain when lips press against the mouthpiece [21]. An alternative to traditional brackets is the Clear Aligners Therapy. These offer greater comfort than traditional braces, as they are less bulky and more likely to prevent irritation, mouth ulcers, and other discomfort associated with orthodontic wires or braces [32]. This option is particularly advantageous for wind instrumentalists, as the aligners can be easily removed while playing. However, more research is needed to evaluate the benefits of Clear Aligners Therapy specifically for wind instrumentalists. 

### 3.4. Temporomandibular Disorder 

TMD is a complex condition that involves the jaw muscles, temporomandibular joints, and adjacent structures of the orofacial area. Any pathological change disrupting their interaction can lead to TMD, commonly expressed as myofascial pain in the fascia and muscles controlling the jaw, neck, and shoulder. Signs and symptoms include headaches, clicking sounds of the jaw, jaw discomfort, finger numbness, and occlusion changes [33]. Traumatic and degenerative disorders are presented to dentists, making diagnosis and treatment challenging for dentists [34]. 

When playing a wind instrument, achieving the perfect embouchure requires the orofacial muscles and temporomandibular joint (TMJ) to work in flawless harmony. However, the physical demands of performing and practicing often lead to muscular imbalances among musicians, particularly in less experienced instrumentalists, who are prone to excessive facial muscle strain [35,36]. This overexertion of the masticatory muscles and the orofacial system may contribute to the onset or exacerbation of temporomandibular disorders (TMD) [16]. For instance, Barros et al. (2018) found that 77% of 30 clarinettists reported experiencing pain in the dentoalveolar components [37]. Additionally, Yasuda et al. (2016) observed higher TMD rates among wind instrumentalists compared to non-wind instrumentalists in a study involving 210 high school students in Japan [38]. 

The incidence of TMD is influenced not only by the type of instrument but also by factors such as playing duration and skill level. Less experienced players, who often exhibit higher facial muscle activity, are at greater risk of losing muscular balance in the temporomandibular system, which can limit mandibular protrusion agility and exacerbate TMD symptoms [19,39]. 

In response to these challenges, Clemente et al. (2018) proposed a diagnostic and treatment scheme for TMD in wind instrumentalists. Using thermograms to analyse muscle activity, they identified higher temperatures on the asymmetrical side, which indicated muscle hypermobility. This hypermobility, often accompanied by pain and discomfort, can hinder both embouchure and performance. The study found that an occlusion splint worn at night decreased TMD symptoms in clarinet players, and specific exercises targeting the facial muscles used in playing were recommended to alleviate TMD pain [22,40]. 

For dentists treating wind instrumentalists with TMD, it is crucial to consider factors such as embouchure, playing duration, and the type of instrument used [17,22]. Although playing a wind instrument may exacerbate or predispose individuals to TMD, it is not necessarily the primary etiologic factor. Various factors can influence the impact of TMJ pain, including susceptibility to stress, emotional profile, sleep quality, socioeconomic background, and the presence of any disabilities.

Despite numerous studies, the relationship between playing wind instruments and TMD remains inconclusive. Notably, Attallah et al. (2014) and van Selms et al. (2017) found no definitive association between the two, highlighting the need for further research with larger sample sizes to better understand the long-term effects of playing wind instruments on TMD [16,17]. 

### 3.5. Oral Mucosal Diseases

Diseases of the oral mucosa caused by microorganisms such as bacteria, viruses, and fungi are not life-threatening but often affect professional wind instrumentalists. These diseases significantly impact oral health and quality of life, with some being highly contagious, requiring careful diagnosis, targeted therapy, and prevention by dentists. 

Various studies confirm that wind musicians’ mouthpieces are frequently contaminated with bacteria, viruses, yeast, or molds. Marshall and Levy (2011) found that most bacterial species survived up to 24–48 h, with attenuated *Mycobacterium tuberculosis* surviving up to 13 days on reeds. Less contamination was found on brass mouthpieces than on reeds. Common bacteria identified included *Streptococcus* sp., *Staphylococcus* sp., *Moraxella* sp., and *E. coli* [41].

After extensive performance, saliva coats the mouthpiece and inner part of the instrument. Viruses such as Hepatitis A and B, Epstein–Barr, and cytomegalovirus can be transmitted via saliva on the mouthpiece [42]. Therefore, sharing mouthpieces is inadvisable, and instruments should be cleaned or decontaminated using alcohol wipes, detergents, or hypochlorite [13].

Steam disinfection is highly recommended as it leaves no bacteria or yeast on the mouthpiece [43]. However, this method can damage reed mouthpieces and is optimal only for brass ones. Despite its high cost and maintenance, regular cleaning of mouthpieces is essential for preventing infections. 

Herpes labialis is more common in brass instrumentalists than in non-wind players [44]. This condition is caused by the constant mechanical stress and vibrations on the lips during performance [14,42]. It results in sore mouth and blisters, reducing comfort and causing pain during play. Although there is no cure for herpes, antiviral medications can quickly prevent outbreaks if noticed early. Dentists should handle herpes-infected areas with care, avoiding stress on the sensitive area, especially at the infection’s onset. They should always wear gloves, masks, and eye protection due to herpes’s high infectivity.

### 3.6. The Respiratory System

The respiratory system (RS), comprising the airways, lungs, and blood vessels, is essential for gas exchange and crucial for professional musicians using vocal cords or instruments. The lungs force air through the airways, into the mouth, and out via the lips into the musician’s mouthpiece.

Few studies have examined the influence of wind instruments on respiratory function. Bouros et al. studied 16 woodwind and 16 brass players, finding that playing wind instruments positively affected respiratory function, with no difference between smokers and nonsmokers [21]. Another study evaluated 99 professional male musicians in Zagreb (Croatia), with an average age of 33 years and 19 years of employment [45]. This study found that the musicians’ lung function was comparable to or better than the controls. However, upper airway symptoms like sinusitis, nasal catarrh, and hoarseness were more common in wind instrumentalists [45].

Infections of the maxillary sinuses are significant to dentists. Maxillary sinusitis, an infection of the paranasal sinuses, is diagnosed either by acute symptoms or imaging techniques (X-ray/digital volume tomography) in chronic cases. Zuskin et al. (2009) found that 22.2% of wind instrumentalists had sinusitis [45]. However, sinusitis can have multiple causes, including flu, viruses, bacteria, allergies, and pulpitis. While wind instruments can affect sinusitis development, they are not the primary cause.

Other infectious diseases are rarely transmitted by the exhaled air from wind instruments. The airflow produced by wind instruments does not significantly affect compacted air, and measurements beyond 1.5 m show no airflow [46]. Thus, the risk of severe acute respiratory syndrome coronavirus-2 infection is minimal; however, constant fresh air is recommended.

The existing research results on lung function and sinusitis are fairly limited, and more studies are required to adequately assess the general effect of wind instruments on RS. Furthermore, a greater number of participants is necessary to provide a suitable statement.

### 3.7. Allergic Reactions in the Mouth Area

Contact dermatitis on a musician’s mouth, primarily caused by the mouthpiece, is a significant health issue. Most brass instruments contain nickel, to which about 4.5% of the population is sensitive [47]. Studies have shown that nickel can cause contact dermatitis on the hands, fingers, lips, and neck [42]. Key symptoms include itching, red rash, blisters, and dry or cracked skin. Gambichler, Boms, and Freitag (2004) recommended that musicians who are allergic to nickel switch to plastic or gold mouthpieces [42]. Woodwind and brass instrumentalists are more likely to develop contact dermatitis compared to string players [42]. Cane reeds, related to bamboo, are used in instruments like saxophones and clarinets, where the musician applies pressure with their teeth and lips. Those allergic to cane reeds should use polystyrene reeds. Dentists should be aware of contact dermatitis in the orofacial region of musicians, as they may encounter such cases. Further research with more patients is needed to fully understand contact dermatitis’s impact on wind instrumentalists.

### 3.8. Orofacial Traumatology

Injuries in the orofacial region of professional wind instrumentalists are crucial as they threaten their ability to perform. Depending on the trauma’s type and severity, these injuries require treatment by specialised dentists, oral surgeons, or maxillofacial surgeons. As mentioned, the anterior teeth are vital for embouchure and stabilising the instrument. Changes in the teeth’s dimension or position cause discomfort and hinder playing, including issues like sharp edges on dentures and insufficient dental fillings. For avulsion of the anterior teeth due to orofacial trauma from a fall, the tooth should be reimplanted within 30 min for the best success rate [48,49]. If immediate reimplantation is not possible, the teeth should be stored in milk or saliva until a dentist is available [50,51]. Other solutions, like powdered baby milk or Hank’s Balanced Salt Solution, are also useful for keeping the tooth cells vital but may not be readily available after trauma [52].

### 3.9. Special Measures

Surgery and tooth extraction are also performed by professional instrumentalists, and dentists should be aware of certain specifics. Intraoral pressure impacts wound healing, so after a simple tooth extraction, patients are advised not to play for two weeks. For more complicated extractions, such as wisdom teeth, a month-long break is recommended [14]. In rare cases, like an oral-antral connection, musicians should wait at least a month before resuming due to the risk of rupturing the Schneider membrane, which can lead to maxillary sinus infection and other issues.

Dentists should also educate patients, especially professional wind instrumentalists, about the risks of local anaesthesia, which can cause self-inflicted injuries if not fully worn off [14]. This is particularly important after inferior alveolar nerve anaesthesia, as the musician may not feel their lips, making them susceptible to injuries from force (e.g., from the mouthpiece) or thermal influences (e.g., hot drinks). While these injuries are usually mild, they can disrupt embouchure and cause painful wounds that require a pause in playing. Therefore, dentists should use minimally invasive procedures and inform musicians about temporary performance limitations during preoperative consultations.

### 3.10. Measures of Prevention

Most problems and effects of wind instruments on orofacial structures can be minimised through preventive measures taught early in music schools and universities. This is especially important as many students are unaware of the orofacial consequences of using wind instruments. Although dental trauma is more common in primary dentition, it has a 20% prevalence in secondary dentition of children and adults [53]. Orofacial injuries at a young age can affect the careers of professional musicians. Most orofacial trauma occurs in children aged 10–14 or during high-risk sports like hockey, American football, polo, rugby, skateboarding, and parkour [11,23]. Dental mouthguards significantly reduce the risk of sports injuries to the chewing apparatus.

Professional wind instrumentalists should be aware of the importance of their teeth and the risks they face, such as bicycle accidents that can cause trauma to the anterior teeth. These unpredictable events make suitable insurance advisable for professional musicians. Additionally, musicians should undergo regular dental check-ups and impressions of their masticatory apparatus [21,23]. While classic plaster casts detail the patient’s dental situation, they are brittle and cannot be used directly. In contrast, intraoral scans create exact three-dimensional models of the teeth, allowing dental technicians to design precise dentures using automated milling systems. These dental impressions or scans are crucial for accurately reconstructing damaged, decayed, or extracted teeth, allowing dentists to treat patients without significantly affecting their embouchure.

Wind instrumentalists experience increased saliva flow when playing, leading to increased plaque and possibly dental calculus formation, which can worsen existing periodontal problems [14,54]. Therefore, wind instrumentalists should visit an oral hygienist 1–2 times per year, depending on the diagnosis.

## 4. Discussion

Based on the results of this article, we conclude that playing a professional wind instrument has a significant impact on the oral health of musicians. To the best of our knowledge, this article represents a current broad-based review of a topic that does not exist in this form. Previous studies have mostly focused on one specific problem (e.g., TMD, RS, or contact dermatitis), and none have examined the overall oral problems of wind musicians.

There are two important perspectives to explore: first, how playing wind instruments can influence oral health, and second, how oral health issues and medical conditions might impact a musician’s ability to play these instruments effectively. To gain a comprehensive understanding of these interactions, it is crucial to conduct in-depth research. Epidemiological studies, along with longitudinal studies that track changes over time, are especially important to thoroughly investigate these relationships and their implications.

Regarding one of the most important fundamental questions of this article, the majority of authors have approved whether oral health can be affected by playing a wind instrument. Only 2 of the more than 30 authors are sceptical about these claims [16,17]. However, both these authors referred primarily to the TMD problem and previous studies. We agree with their opinion and are convinced that clear conclusions can only be drawn if systematic reviews or meta-analyses on this topic are available. However, expert interviews clarified that some professional wind instrumentalists experience pain and TMD when playing intensively over many years.

### Strengths and Limitations

The results presented clearly demonstrate that a significant proportion of oral diseases occur at a higher rate among professional wind musicians compared to the general population. However, many studies used small sample sizes, making it difficult to draw definitive conclusions. As previously mentioned, it is almost impossible to find a large, statistically representative group of professional instrumentalists who play the same instrument, which can be studied over a defined period of time and experience similar problems. This situation severely limits the use of many studies but provides evidence and arguments for a better understanding of orofacial problems in wind instrumentalists. Further studies are needed on all facets of the subjects evaluated in this study. In this article, the limitations of many studies highlight the need for further research on all aspects of oral health among wind instrumentalists. An interdisciplinary approach is necessary, with interest and participation from both dentists and professional musicians or their representatives or professional associations.

The interviews were beneficial for continuing the discussion on the topic, as they provided a contrast between the perspectives of dentists and patients. However, the shared experiences and observations are only representative of one individual’s point of view. To gain a more comprehensive understanding of the issue, it would be beneficial to conduct standardised long-term studies and systematic interviews with members of large ensembles, going beyond the individual. This would provide more relevant information on the oral health of wind musicians.

In future studies, it will be essential to include questionnaires, clinical examinations, and psychoemotional assessments of musicians. These components will provide a comprehensive understanding of the various factors influencing both oral health and musical performance.

Important supplementary information for evaluating the health status and occupational diseases of professional musicians can be obtained from data provided by professional insurance companies, health insurance providers, and professional associations of this occupational group.

## 5. Conclusions

The findings of this study offer a novel perspective on the orofacial challenges encountered by wind instrumentalists, emphasising the critical role of the teeth, lips, mouth, and jaw in instrumental performance. While the spectrum of oral diseases may not be widely recognised among young musicians, its profound impact on labour, social, and health policy aspects cannot be understated. Our research underscores the necessity for further investigation to validate and expand upon these insights, particularly through long-term studies that delve deeper into the implications. These findings hold significant relevance for a diverse range of stakeholders, including wind instrumentalists themselves, dental practitioners, music educators, healthcare providers, and insurance companies. By recognising and addressing these oral health issues, we can enhance the well-being and longevity of wind musicians while fostering a broader understanding of the intersection between music performance and oral health.

## Data Availability

The datasets generated and analyzed during the current study are available in the Supplementary Materials.

## Supplementary Materials

The following supporting information can be downloaded at: https://www.mdpi.com/article/10.3390/dj12100306/s1, Supplementary Table S1. S1_Table [4,5,6,7,10,12,13,14,15,16,17,18,19,20,21,22,23,25,27,30,36,38,39,40,41,43,46,47,55,56,57,58,59,60,61,62,63].
