# Supplementary material for: Wind Instruments and Oral Health: Challenges Faced by Professional Wind Musicians
Source: Dent J (Basel). 2024 Sep 26;12(10):306. doi: 10.3390/dj12100306 (PMC11505960; doi:10.3390/dj12100306)
Supplement: Supplementary file 1 [file dentistry-12-00306-s001.zip › dentistry-3093149-supplementary.pdf]

Supplementary Table S1:

No.	Author	Year published	Objective	Type of Study	Sample Size	Intervention	Outcomes/Results
1	Okoshi et al.	2017	To examine the different health issues associated with playing musical instruments	Review	n.a.	・Various articles and studies were evaluated	・There are numerous health issues associated with playing music instruments<br>・None are serious or fatal
2	Yeo et al.	2002	To give an insight into specific orofacial problems and give guidance to general practice dentists	Review	・Studies published in dental, music and health journals	n.a.	・Musicians experience a varielty of orofacial problems<br>・Musical instruments may assist tooth movement during orthodontic treatment<br>・Focal dystonic is uncommon; however, it may result in an loss of embouchure control<br>・Dental prostheses intrude the embouchure<br>・Herpes labialis is common during times of performance and stress<br>・Patients should not play wind instruments after local anaesthesia or after extractions
3	Glowacka et al.	2014	To assess if string and wind instruments influence the stomatognathic system	Review 10/10	n.a.	n.a.	・Long-term playing causes craniomandibular dysfunctions and 80% of the symptoms can be reduced by orthodontic plates<br>・Playing the violin causes malocclusion due to changes in bone growth
4	van der Weijden	2020	To determine if wind instruments influence the tooth position or facial morphology	Review 10/221	・Sample size from 36–170 participants<br>・Child - professional musicians	・PubMed<br>・EMBASE<br>・Cochrane-CETRAL<br>・Orthodontic journals<br>・Google Scholar	・Wind instruments may influence the facial morphology and tooth position of adults and children<br>・Larger overjet is common in adults playing single-reed instruments<br>・Increased maxillary and mandibular intermolar width is associated with brass players<br>・Thicker lips are common in children playing wind instruments<br>・Significant reduction in overjet for children playing brass instruments
5	Attallah et al.	2014	To determine the association between TMD and playing musical instruments	Review 14/19	n.a.	・Mesh search in National Library of Medicine's Medline Database	・A potential association between playing wind instruments and TMD<br>・Violin and viola players are especially associated with TMD<br>・Increase in awareness to seek better treatment
6	Barbieri et al.	2020	To assess the impact of playing wind instruments on oral conditions	Review 18/46	n.a.	・PubMed<br>・Likacs<br>・Scielo<br>・Cochrane<br>・Google Scholar	・Musicians should clean their instruments after use with alcohol wipes or swabs to disinfect their mouthpiece and reed<br>・Instruments can influence the tooth position
7	van Selms et al.	2017	To investigate the effect of wind instruments, violin, viola, and singing on TMD	Review 15/54	n.a.	・Mesh search in National Library of Medicine's Medline Database<br>・PubMed search<br>・Web of Science<br>・Google Scholar<br>・Risk of bias assessment using a modified Newcastle-Ottawa Scale (NOS) (Scale 0–7)	・Median NOS score was three points<br>・Weak evidence, that music instruments are an risk factor in developing TMD<br>・Vocalists did not experience more TMD that controls<br>・Signs and symptoms associated with TMD have been found in studies which performed clinical examinations in viola and violin players
8	Macovei et al.	2023	To assess the impact of wind instruments on Dento-Facial Morphology	Review 53/295	n.a.	・PubMed search<br>・Research Gate<br>・Google Scholar<br>・Websites<br>・Organisations<br>・Speciality Books	・Less experienced instrumentalists suffer from excessive strain due to increased facial muscle engagement<br>・A series of exercises for overloaded muscles was identified<br>・Lip tension has a great impact on tooth movement
1	Adeyemi and Otuyemi	2019	To evaluate the effect of music instruments on occlusal characteristics in northern Nigerian population	Original article	・50 male wind musicians between the age of 18–45 years, playing wind instruments for a minimum of 2 years<br>・Full dentition<br>・50 controls	・Questionnaire<br>・Dental casts<br>・Digital calliper for arch dimensions	・Playing wind instrument significantly affects the alignment of the anterior-segment<br>・The occlusal characteristics are not significantly affected by wind instruments

2	Clemente et al.	2020	To determine a concept of oral rehabilitation of an orofacial trauma	Original article	・13-year old boy playing the trumpet<br>・Bike accident with the avulsion of tooth 11 and 21 at the site	・Panoramic radiographic examination<br>・Extra-oral palpation<br>・Extraoral photographs<br>・Hawley appliance<br>・Trumpet edentulous anterior tooth appliance<br>・Splint<br>・Microphone recordings	・Intra-oral appliances suit the patient best in terms of sound quality, comfort and aesthetic<br>・Dental impressions should me made of young wind instrumentalists, to have the most reliable anatomy in case of a trauma<br>・The smaller the appliance, the greater the degree of control
3	van Selms et al.	2019	Risk indicators and prevalence for TMD for musicians	Original article	・90 music ensembles with musicians over the age of 18 years	・Questionnaire	・1,470 musicians completed the questionnaire<br>・Response rate: 77%<br>・Mean age: 41.6 years<br>・46.5% male<br>・18.3% of musicians reported TMD pain<br>・Upper string players (69.2%) most likely to have neck and shoulder pain<br>・"Woodwind players, having a younger age, showing loss of interest and having adverse oral behaviours were associated with higher odds for having TMD pain"<br>・TMD pain may be associated with playing woodwind instruments<br>・Pain in neck and shoulders is associated with violin/viola players
4	Clemente et al.	2018	To describe the steps in the diagnosis and treatment of wind instrumentalists with TMD	Original article	・30-year old female Caucasian clarinettist	・Questionnaire<br>・Clinical examination<br>・Opening pattern<br>・Eccentric movement<br>・Occlusional analysis<br>・Extra-oral analysis<br>・Thermal imaging infrared camera<br>・Piezoresistive sensor	・Occlusal splint was used at night while sleeping, for 6 months<br>・Occlusional splint allowed a reduction in muscle activity and TMD symptoms<br>・Infrared thermograms and piezoresistive sensors can be used for diagnosis of TMD
5	Nishiyama and Tsuchida	2016	To assess the risk of TMD among wind instrumentalists	Original article	・72 wind instrumentalists younger than 30 years<br>・playing for minimum of 1 year<br>・66 non-players<br>・51 men and 87 women	・Questionnaire	・Greater prevalence (29.1%) of TMD in the instrumentalists compared to the control<br>・Mouthpiece pressure was a significant factor contributing to TMD<br>・Correct embouchure is of importance
6	Adeyemi and Otuyemi	2020	Assessment of effects of playing music instruments in male Nigerian adults on TMJ	Original article	・50 men between the age of 18 and 45 years, playing wind instruments for a minimum of 2 years<br>・50 controls	・Questionnaire	・Significantly higher TMJ dysfunction prevalence in wind instrumentalists compared to control group<br>・Slightly higher anamnestic score in clarinet/saxophone players<br>・TMJ dysfunction is significantly affected by playing wind instruments, especially clarinet/saxophone
8	Pampel et al.	2014	To examine if playing wind instruments contributes to TMD	Original article	・102 men aged ≥ 18 years<br>・33 wind musicians (18 professionals, 15 amateurs)<br>・33 patients with TMD<br>・36 healthy participants	・Physical examination<br>・Inspection<br>・Questionnaire	・All examined wind instrumentalists have one or more parafunctional habits<br>・Wind instrumentalists are at a higher risk of loosing muscular balance in the TMS<br>・No clear correlation between playing technique and TMD
	Yasuda et al.	2016	To examine if playing music instruments have an effect on muculoskeltal functions of high school students	Original article	・210 high school students playing wind instruments<br>・35 boys<br>・175 girls<br>・>6 days/week practice<br>・Mean age: 14 years	・Questionnaire<br>・Muscle activity in musculus temporalis, masseter, sternocleidomastoid and trapezius while playing (11 boys and 37 girls, with mean age of 13.3 years)	・Overall TMD prevalence of 89%<br>・Wind instrumentalists had a significantly higher rate of TMD (34.8%) compared to non-wind instrumentalists<br>・Duration of playing wood-wind instruments with a reed mouthpiece and brass-wind instruments with a small mouthpiece affects TMD<br>・Prevalence is higher in wood-wind and brass-wind instrumentalists than in in no-wind instrumentalists
9	Bouros et al.	2018	To examine the function of respiratory system in wind instrumentalists	Original article	・33 male and 9 female professional wind instrumentalists<br>・Aged 20–53 years old<br>・Mean age: 32.69 years	・Questionnaire<br>・Cosmed's Pony spirometer	・33 participated (23 males and 9 females)<br>・No significant sign of strain of the respiratory system after rehearsal<br>・Respiratory functions of smokers and nonsmokers showed no differences after rehearsal
10	Zuskin et al.	2009	Investigation on the effect of playing wind instruments on lung function and the respiratory system	Original article	・99 male professional wind instrument players<br>・Aged 21–61 years<br>Mean age: 35 years<br>・36 smokers smoking an average of 20 packs/year<br>・41 string instrumentalists as controls	・Questionnaire<br>・Spirometer Pneumoscreen	・Wind instrumentalists have a higher prevalence of upper airway symptoms (sinusitis, nasal catarrh and hoarseness)<br>・Musicians who worked for the longest time, showed higher lung function measurements<br>・Playing wind instruments may be associated with higher than expected lung function parameters

**11**	Spahn et al.	2021	To evaluate if the airflow in wind instruments influences the risk of SARS-CoV-2 Infection	Original article	14 professional wind instrument players	・Camera<br>・Flow-MakerTM<br>・SAFEX (artificial fog)<br>・Low Velocity Flow Analyser	・No airflow escaping from any part of the different instruments was measurable beyond a distance of 1.5 m<br>・The pitch and volume did not influence the results
**12**	Gambichler et al.	2004	To evaluate the effect of music instruments on skin conditions and contact dermatitis	Original article	n.a.	・PubMed<br>・National Library of Medicine's Medline Database<br>・Life science journals	・There are a number of skin conditions and contact allergies regarding music instrumentalists<br>・Herpes labiales is common in woodwind and brass instrumentalists<br>・Contact allergies mainly include: colophony, nickel and exotic woods
**13**	Woldendrop et al.	2016	To provide a narrative review on the fundamentals of brass player's embouchure	Original article	n.a.	・National Library of Medicine's Medline Database<br>・Cochrane<br>・Web of Science<br>・CINAHL database<br>・Oxford Music Online<br>・Geschichte und Gegenwart	・New definition of embouchure: embouchure is the process needed to adjust the amount, pressure,<br>and direction of the air flow (generated by the breath support) as<br>it travels through the mouth cavity and between the lips, by the<br>position and/or movements of the tongue, teeth, jaws, cheeks,<br>and lips, to produce a tone in a wind instrument"<br>・Physics of embouchure: Venturi's and Bernoulli's principle<br>・Embouchure related structures: lips, teeth, tongue, cheeks and jaw
**14**	Barros et al.	2018	To analyse the orofacial structures using a thermographic camera in clarinet players	Original article	・30 professional clarinettists<br>・Practice: minimum 2h daily<br>・Age: 18 to 49 years<br>・Mean age: 25.5 years	・Photographs (intra- and extra-oral)<br>・Thermometer TESTO 175-H1 for ambient conditions<br>・Thermographic images using Flir E60s camera	・Cranio-cervical-mandibular complex can be successfully diagnosed using infrared thermography<br>・Statistical significant difference in left temporal muscle, orbicularis muscle, left and right perioral teguments<br>・76% reported pain in teeth 11 und 21<br>・Measuring the muscle activity provides a better understanding of the embouchure and later treatment options
**15**	Franz et al.	2020	To evaluate the facial muscle patterns in clarinet players and understanding the physiology and dysfunction	Original article	・8 professional clarinettists<br>・4 female students, mean age: 20 years<br>・4 postgraduate students/teachers, mean age: 33 years (3 males, 1 female)	・Surface electromyographic measures<br>・Muscles measured: sternocleidomastoid, masseter, mentalis, mylohyoid and buccinator muscles	・Less experienced group showed a significantly higher facial muscle activity (buccinator, masseter and mylohyoid muscles)<br>・Experienced players showed optimised muscle activity
**16**	Iltis et al.	2019	To evaluate how embouchure dystonia affects tongue position variability in dystonic and healthy horn players	Original article	・14 French hornists<br>・9 healthy elite players<br>・5 elite players diagnosed with embouchure dystonia<br>・1 musician with active international solo career<br>・3 professors of horn	・A sustained Eb4 pitch for 6 s at 3 dynamic levels<br>・MRI film at 33.3 ms resolution	・Tongue position variability and the oral cavity differed with different dynamics for all participants<br>・Elite players have a greater tongue position variability
**17**	Clemente et al.	2019	To evaluate if piezoresistive sensors are a valid measuring tool to measure the forces applied by the mouthpiece on the lower lip	Original article	・5 clarinet players<br>・5 saxophone players	・Piezoresistive sensor<br>・3 different notes performed (high-, medium- and low-pitch).	・Values for clarinetists: 16–226 g<br>・Values for saxophonists: 5–320 g<br>・Forces applied to the lower lip are equivalent to medium orthodontic forces<br>・Lip pressure appliances are valid treatment solution
**18**	Herman et al.	1981	To determine if music instruments cause tooth movement in junior high school children	Original article	・5 junior high schools<br>・Began with 220 instrumentalists and ended with 91 instrumentalists<br>・56 students and ended with 36 controls<br>・Aged 11–13 years<br>・Mean practice time, year 1: 104 min<br>・Mean practice time, year 2: 111 min	・Questionnaire<br>・Interview<br>・Oral exam with angle classifications<br>・Dental casts	・Statistically significant anterior tooth movement was recorded for almost all musicians, minimum movement recorded for the control group<br>・Dentists can suggest different music instruments to help influence the kids overbite/overjet and certain tooth movements

**19**	Masiulytė et al.	2021	To evaluate orthodontic problems in string and wind instrumentalists	Original article	・167 students aged 8–25 years<br>・Mean age: 14.59 years<br>・67 males and 100 females<br>・52 string players<br>・46 wind players<br>・69 piano players as control group	・Clinical investigation (ICON index)<br>・Facial profile<br>・Questionnaire	・Common feature of string instrumentalists is the crossbite<br>・Deep bite may be predisposed in wind instrumentalists<br>・Wind instrumentalists showed a frequent overlapping in incisors 13 and 23<br>・61.7% of string students had a crossbite
**20**	Clemente et al.	2021	To examine the tooth position in wind instrumentalists using a cephalometric analysis	Original article	・48 wind instrumentalists<br>・24 string instrumentalists<br>・Aged 18–40 years<br>・32 women and 40 men<br>・More than 10 years of experience<br>・Exclusion criteria: orthodontic treatment, maxillofacial surgery or mandibular injuries	・Lateral cephalograms	・The interincisal angle showed a statistical significant difference and was higher for the wind group (127.46°) compared to the string group (122.34°)<br>・The inclination of anterior teeth is influenced by the forces during the embouchure
**21**	Lozano et al.	2008	To examine if bruxism is related to violin players	Original article	・41 professional violinists or students in the last 2 years of professional training<br>・Age: 14–47 years<br>・Average age: 33.21 years<br>・20 men and 21 women<br>・50 healthy controls (17 males and 33 females)<br>・Exclusion criteria: TMD or orthodontic treatment	・Questionnaire	・Teeth grinding and clenching statistically more common in violinists<br>・No statistically significant difference between men and women and bruxism<br>・No statistically significant difference in no. of hours of practice and and bruxism
**22**	Kula et al.	2015	To determine if malocclusion is associated with trumpet performance skills	Original article	・70 university students of trumpet (54 males, 16 females)<br>・Mean age: 22.2 years<br>・From 11 universities	・3D cone-beam computerized tomography scan	・Reliability exceeds an excellent value of r=0.94<br>・Positive association between arch widths and various skills<br>・Negative association between maxillary central incisor rotation, Little's irregularity index, articulation performance and interincisal inclination
**23**	Clemente et al.	2019	To determine a new classification of wind instruments	Original article	・38 professional musicians<br>・5 clarinet, 1 oboe, 4 bassoon, 6 trumpet, 4 French horn, 4 tuba, 4 trombone, 7 saxophone, 2 transversal flute and 1 flute player<br>・Exclusion criteria: no permanent dentition and orthodontic treatment	・Radiographic examination<br>・Cephalometric tracing	・Saxophone mouthpiece should enter the mouth at approximately 20°<br>・The oboe has a close to vertical playing position inside the mouth<br>・The larger the mouthpiece, the lower the sound<br>・Teeth, lips and Dental arches are of great importance at a new nomenclature and classification
**24**	Clemente et al.	2020	To examine the association between dental the interarch relationship and wind or string instruments	Original article	・77 orchestra musicians and university students<br>・50 wind instrumentalists<br>・27 string instrumentalists<br>・More than 10 years of experience, no orthodontic treatment and no maxillofacial surgery or mandibular injuries	・Lateral cephalograms	・66% of wind instrumentalists showed a Class II malocclusion<br>・Wind instrumentalists showed a statistically significant difference in the position of their lower incisors, as they were more orthopositioned<br>・Wind instruments have an insignificant craniofacial influence<br>・Winding instruments may have little dentofacial influence
**25**	Adeyemi and Otuyemi	2020	To determine the difference in dental arch dimensions in wind- and non-wind instrumentalists	Original article	・50 males musicians between the age of 18 and 45 years, playing wind instruments for a minimum of 2 years<br>・50 controls<br>・Full dentition	・Dental casts<br>・Digital calliper for arch dimensions	・The arc dimensions were not affected significantly by type of instrument, duration and frequency played
**26**	van der Weijden	2018	To determine if wind instruments performance and embouchure comfort is influenced by tooth position	Original article	・Sample size: 20–100 participants	・PubMed<br>・Cochrane<br>・Embase<br>・Google Scholar<br>・Orthodontic Journals	・Tooth position can be influenced embouchure comfort and musical performance according to four studies<br>・A Class I without malocclusion suits all wind instruments<br>・The greater the malocclusion, the bigger the interference with embouchure comfort
**1**	van der Weijden et al.	2019	To describe how orthodontic tooth corrections may improve the embouchure	Case study	・48-year old professional male horn player with embouchure problems	・Plaster models<br>・Clinical pictures<br>・Orthodontic brackets<br>・Composite veneer<br>・Fixed retainer	・Lower anterior teeth were aligned using orthodontic brackets and a 0.018 NiTi wire<br>・Upper front teeth were regulated using composite veneers<br>・Hornist experienced improvements in embouchure<br>・Orthodontic treatment can improve the embouchure but upper retainers and lingual brackets may be disturbing

**2**	Gunst et al.	2011	To evaluate if external cervical resorption is a result of a pressure trauma by frequently playing wind instruments	Case study	・Case 1: 38-year old African male suspected of invasive external cervical resorption on 3 molars<br>・Case 2: 41-year old Caucasian female with a 'darkening pink spot in the cervical area'	・Cone-beam computer tomography (CT)<br>・Micro-focus CT<br>・Photos using a Camera<br>・Probing<br>・Embouchure analysis	・The pressure generated by wind instruments may be a factor of external cervical resorption<br>・ External cervical resorption is still poorly understood
**3**	Shafi and Welbury	2015	To investigate if apical root resorption is caused by playing instruments	Case study	・Case 1: 18-year old girl with shortened radiographic appearance<br>・Case 2: 15-year old girl who fell with her trumpet in her mouth<br>・Case 3: 11.5-year old female who suffered a clash of the head at the playground	・Periapical radiographs<br>・Thermoplastic appliance	・Adequate forces are produced by wind instruments to deflect teeth<br>・Vibration forces increases the risk of root resorption
**1**	Mauersberger	2016	To evaluate the lip vibrations, tone production, and morphological findings of wind instrumentalists	Doctoral Dissertation	・Preliminary study: 13 brass players (23–68 years)<br>・Main Study: 50 brass players<br>・Age: 17–62 years<br>・1h of practice	・Macroscopic clinical judgement<br>・Kymographic and stroboscopic analysis<br>・Rating by: European Laryngological Society<br>・Sonography<br>・Photo documentary	・13/50 instrumentalists showed morphologic changes<br>・7/50 had irreversible scar tissue<br>・1/50 trumpet player had an early stage of scar tissue<br>・3/50 had a swollen lip occupational disability<br>・1/50 had a wollen lymph nodes<br>・1/50 had a midface asymmetry<br>・2/50 had seviere scars on their upper lip diagnosing a occupational disability<br>・Nerve damage due to pressure in the lip area can be seen as an occupational disability<br>・Downstream-Type (98%)
